# Focused Impedance Method (FIM-6) in localized lung ventilation study of the human body in a local setting

**DOI:** 10.2478/joeb-2025-0002

**Published:** 2025-02-19

**Authors:** Trilochan Khanal

**Affiliations:** Department of Physics, Tribhuvan University, Nepal

**Keywords:** Six electrode impedance, lung ventilation study, focused transfer impedance, indigenous technology

## Abstract

The Focused Impedance method (FIM), an innovation by Dhaka University, Bangladesh, is a new technique for focusing a region of interest of a volume conductor through a simple enhancement of the age-old Tetra-Polar Impedance Method (TPIM). This innovation has potential in the diagnosis of different kinds of physiological disorders.

This paper presents the study of lung ventilation on different human subjects using the six-electrode version of the Focused Impedance Method (FIM-6) with the circuit indigenously designed in Nepal. The study was carried out for different quadrants of the lungs of three normal male subjects using both TPIM and FIM-6 configurations, measuring the percentage change in transfer impedance between full inspiration and full expiration. The percentage changes observed were in the range of 15% to 27%. However, errors are expected due to movement of the heart and other organs between inspiration and expiration, which may be difficult to eliminate.

## Introduction

**Fig. 1:**
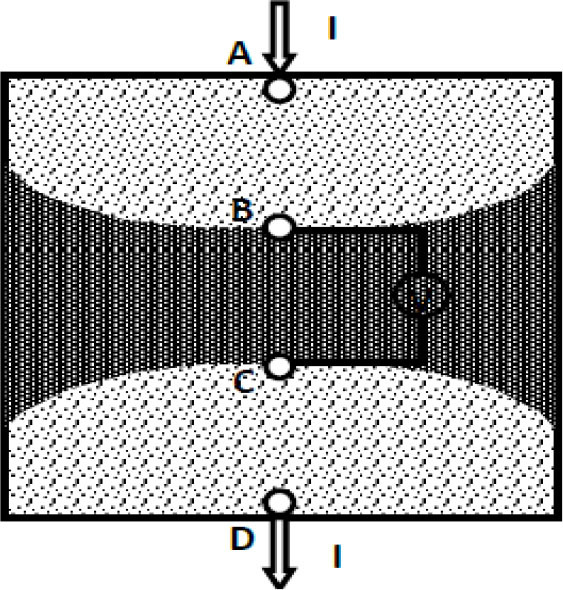
Tetra-polar impedance measurement scheme. From [1] by permission.

A tetra-polar impedance measurement involves the injection of current in a volume conductor through two electrodes (current electrodes) and measuring the resulting potential drop across another pair of electrodes (potential electrodes), as shown in Figure 1. Here, typically a constant current ‘I’ is injected through current electrodes ‘A’ and ‘D’ and potential ‘V’ is measured through the inner potential electrodes ‘B’ and ‘C’, as shown in the figure. The ratio of measured voltage to applied constant current is called Transfer Impedance ‘TZ’ [1]. The advantage of tetra-polar impedance measurement over the standard bipolar or two-electrode technique (based on Ohm’s law) is that TPIM eliminates the effect of tissue-electrode contact impedance.

**Fig. 2:**
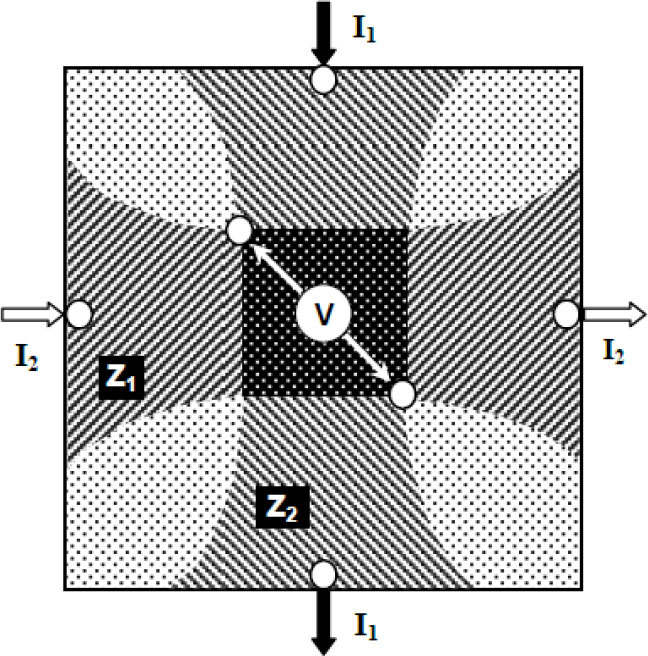
Basic concept of FIM-6. From [2] by permission.

The Focused Impedance Method (FIM) developed at Dhaka University uses 8, 6, or 4 electrodes in its three versions to localize a small zone in a volume conductor. The 6-electrode FIM configuration (FIM-6) is shown in Figure 2 for a two-dimensional conductor [1]. Here, small white circles indicate the electrodes. Two alternating currents, I_1_ and I_2_, are driven between two electrode pairs arranged vertically and horizontally, respectively, as shown in the figure. Two electrodes for measuring potential (potential electrodes) are placed at intermediate diagonal positions, which are the intersection points of the respective equipotential lines due to the two currents in perpendicular directions, as shown in the figure. The potentials V_1_ and V_2_ measured across these potential electrodes are due to the currents I_1_ and I_2_, which individually give the potential differences between the two pairs of equipotential lines shown by the edges of the horizontally shaded region and vertically shaded region, respectively. Furthermore, the values V_1_/I_1_ and V_2_/I_2_ essentially give the Transfer Impedance TZ_1_ and TZ_2_ of the horizontal shaded region and vertical shaded region, respectively, though the contribution of different sub-volumes within this region will vary.

Now if the two currents (I_1_ and I_2_) are electrically isolated but have the same phase (coherent), magnitude and frequency, then the measured potential will essentially give a summation of the two transfer impedances FTZ = TZ_1_ + TZ_2_, which will have enhanced contribution from the dark shaded region in the center. This resultant impedance FTZ is then called Focused Impedance [2,3]. This will hereafter simply be referred to as FZ.

Lung ventilation refers to the exchange of air between the lungs and the environment, which is caused by expansion and compression of the lungs [4]. In various diseases like pulmonary edema and pneumonia, specific regions within the lungs are filled with fluids restricting the air to reach the alveoli, resulting in poor lung aeration. In that case the lung region is said not to be properly ventilated. The electrical properties of lung tissue changes as a function of the air content. By knowing the impedance values of the lungs, the physiological condition can be assessed. The percentage change in FZ during inspiration and expiration is basically a ventilation parameter of the lungs in the respective focused region. With skin surface electrodes, one also gets contribution from the bulk down to a certain depth because of the distribution of the currents inside [5].

This paper presents the assessment of localized lung ventilation on human subjects using the six-electrode version of FIM with the circuit designed indigenously in Nepal [6, 7]. A comparative study of the localizing effect of the conventional Tetrapolar Impedance Measurement System (TPIM) and FIM-6 is also presented.

## Materials and methods

After careful study of the indigenously designed circuit for FIM-6 in a phantom to verify its efficacy, it was employed on human subjects for the lung ventilation study. TPIM measurements were also performed for comparison.

### Linear TPIM measurement

**Fig. 3:**
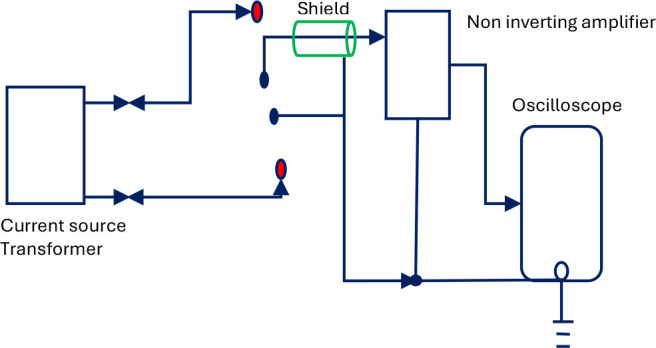
Block diagram for TPIM configuration.

The circuitry used is shown in Figure 3. The outer red circled dots are the current electrodes while the middle blue circled dots are the potential electrodes. The current source transformer (TR1) used for the study had 15 turns in the primary coil and 60 turns in the secondary coil. The actual current passing through the human body was estimated by placing a 55 ohms resistor in series with the secondary coil of TR1, which was removed during actual measurements on the human body. A metal sheet, to be attached to a suitable place on the body of the subject, was connected to circuit ground along with the power supply ground to minimize noise due to 50 Hz AC mains supply.

**Fig. 4:**
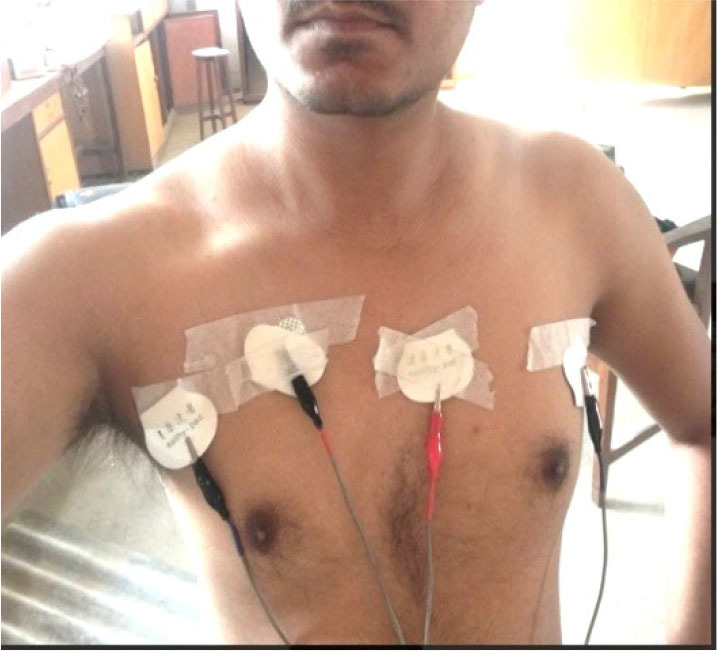
Electrode placement for linear TPIM measurement for lung ventilation.

Figure 4 shows the electrode placement for linear TPIM around the thorax region of a subject. Proper connections with the skin surface were assured using special carbon coated ECG electrodes. The input to the non-inverting amplifier with AC coupling had a shielding. The amplifier gain was set at around 100. An RC coupled high pass filter with cut-off at 1.5 kHz was configured at the non-inverting input of the opamp and a low pass filter with cut off at 50 kHz was configured at the output of the amplifier to reduce noise. The electrodes were placed around the lung region and measurements were carried out at full inspiration and full expiration.

The measurements were carried out while changing the separation between the middle pair of potential electrodes shown in Figure 4. The positions of the outer current drive electrodes were kept the same. At first, the separation between the potential electrodes was set at 10 cm. The transfer impedances were then obtained for the maneuvers of breathe in and breathe out. Then, the separation between the potential electrodes was increased to 15 cm and the same maneuver was repeated for the measurements.

### FIM-6 arrangement

**Fig. 5:**
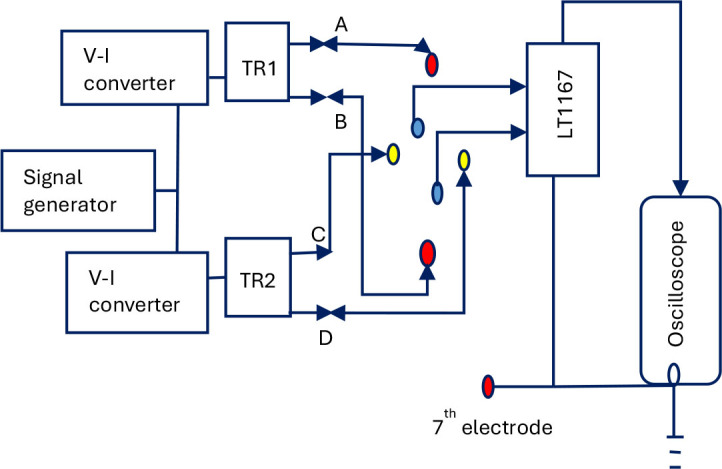
Block diagram for the arrangement of the FIM-6 system.

The circuit for FIM-6 follows the original design from 1999 by Rabbani et al. [3]. As mentioned in the previous section this arrangement needs concentric and orthogonal current drives using two pairs of electrodes, and the potential is measured using a diagonal electrode pair, which is also concentric with the current electrodes. A basic block diagram of the relevant parts of the FIM-6 circuit used for the present work is shown in Figure 5. Here, the outer red dots represent one pair of current electrodes while the yellow dots represent the electrodes of the orthogonal current drive. The two blue dots in the middle represent the diagonal potential electrodes. If the alternating currents through both the current drives are maintained constant at the same value, I, and if the potential measured across the middle electrodes be V, then the focused impedance, FZ is given by,

(1) FZ=V/I

This arrangement requires that the two current drives are electrically isolated but their driving signals are coherent and have the same phase. To achieve this, the same signal generator is used to drive both orthogonal current drive electrode pairs. However, these need to be electrically isolated so that the two orthogonal current distributions maintain their individual distribution patterns within the volume conductor, but the potential developed across the potential electrode pair has contributions from both the currents. Through a suitable choice of the polarity of the transformer, this arrangement can essentially produce two independent current sources that are coherent.

The middle two potential electrodes were connected to the two differential inputs of the instrumentation amplifier IC (LT1167) as shown in Figure 5. A 7^th^ electrode was used as the reference electrode for potential measurements.

To verify if we are getting the same current when connected to a human body, a direct measurement was made before the actual experiments.

For this, a 56 ohms resistor (actual measured value: 55.4 ohms) was connected in series between the secondary windings of one transformer and a pair of current electrodes fixed on the body. Typically, the total impedance offered by the two electrodes connected to the body would be of the order of a thousand ohms. Therefore, 56 ohms will not change the current significantly. The voltage across the series resistor was measured using an oscilloscope at both breathe in and breathe out conditions, which when divided by 55.4 ohms essentially gave the actual current passing through the body. For this measurement, the probe and ground electrodes of the oscilloscope were connected to the secondary windings of the respective transformer. Then a similar measurement was performed for the other current channel (through the other transformer) and the other pair of current electrodes. For a perfect current source, the above voltage should remain the same for all the above conditions, for breathe in and breathe out conditions and for both the channels. However, it is very difficult to make a perfect current source, so there may be some variations, which will give rise to some errors. The aim should be to minimize such errors.

**Fig. 6:**
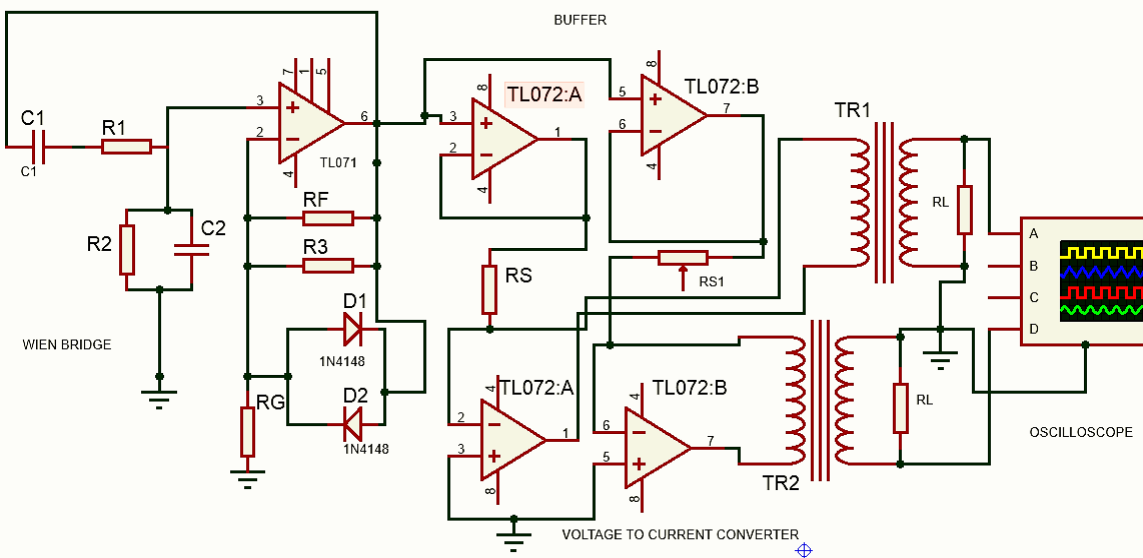
Circuit for the current drive section for the FIM-6 system designed in the Proteus software (Labcenter Electronics Ltd, England) [7].

The circuit used for the two sets of orthogonal current drives is shown in Figure 6. The first section on the left is a Wien bridge oscillator producing a sinusoidal alternating voltage of a chosen frequency and amplitude. This is then taken to two unity gain buffers the outputs of which are used to drive two separate voltage-to-current converter circuits. Here the load seen by each voltage-to-current converter circuit essentially is a compound one, consisting of the final load at the secondary windings of the current transformer. This compound load is connected between the output and the inverting input of the respective opamp. The currents through the two loads at the secondary outputs of the two transformers are balanced using series resistors R_S_ and R_S1_ from the outputs of the buffers to the inverting inputs of the two opamps that make the voltage-to-current converter circuits.

In order to make both the final load currents the same, R_S_ is given a fixed value of 1.00 kΩ while R_S1_ is varied till the load currents of the two current outputs are the same. For the load, resistors with a value of 3 kΩ each were connected at the outputs of the secondary windings of the transformers. For this load current measurement, one terminal of each transformer was shorted to the oscilloscope ground and the voltages at the other ends of the load resistors were measured using the two channels of the oscilloscope. R_S1_ was then adjusted to get the same voltage on both the outputs, which effectively gave the same load current through both the load resistors. When applied to a human body, the load resistors may not be exactly equal and may not have a value of 3 kΩ. However, since a voltage-to-current converter circuit drives the same current for a reasonable variation in the load resistance, we may expect to have constant currents when applied to a human body.

**Fig. 7:**
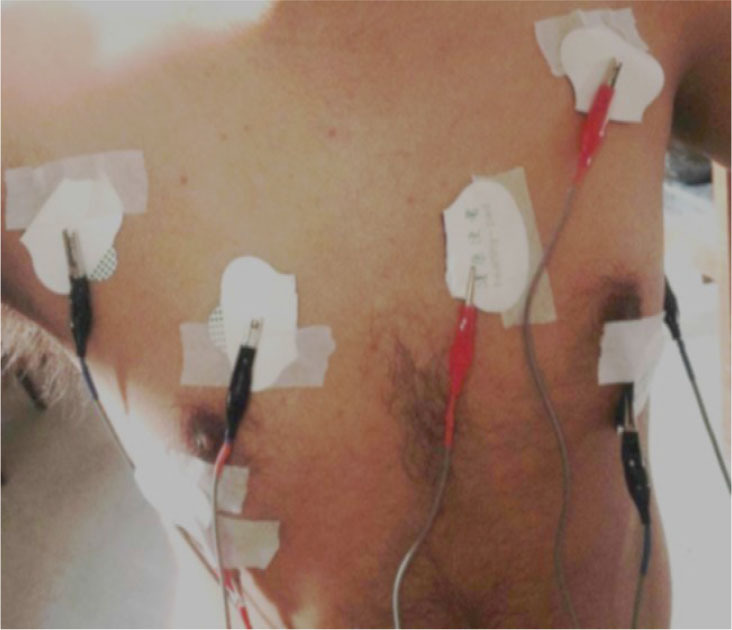
Electrode placement for FIM-6 for measurement at the mid-region of the chest.

Figure 7 shows the placement of electrodes on a human subject for measurement at the mid region of the chest. The four outer ones form the two orthogonal current drive pairs while the middle pair is for potential measurement. The current electrode positions were kept fixed with separations of 30.0 cm between the electrodes of each pair. Measurements were carried out for two separations of the potential electrodes, 10.0 cm and 12.5 cm respectively.

**Fig. 8:**
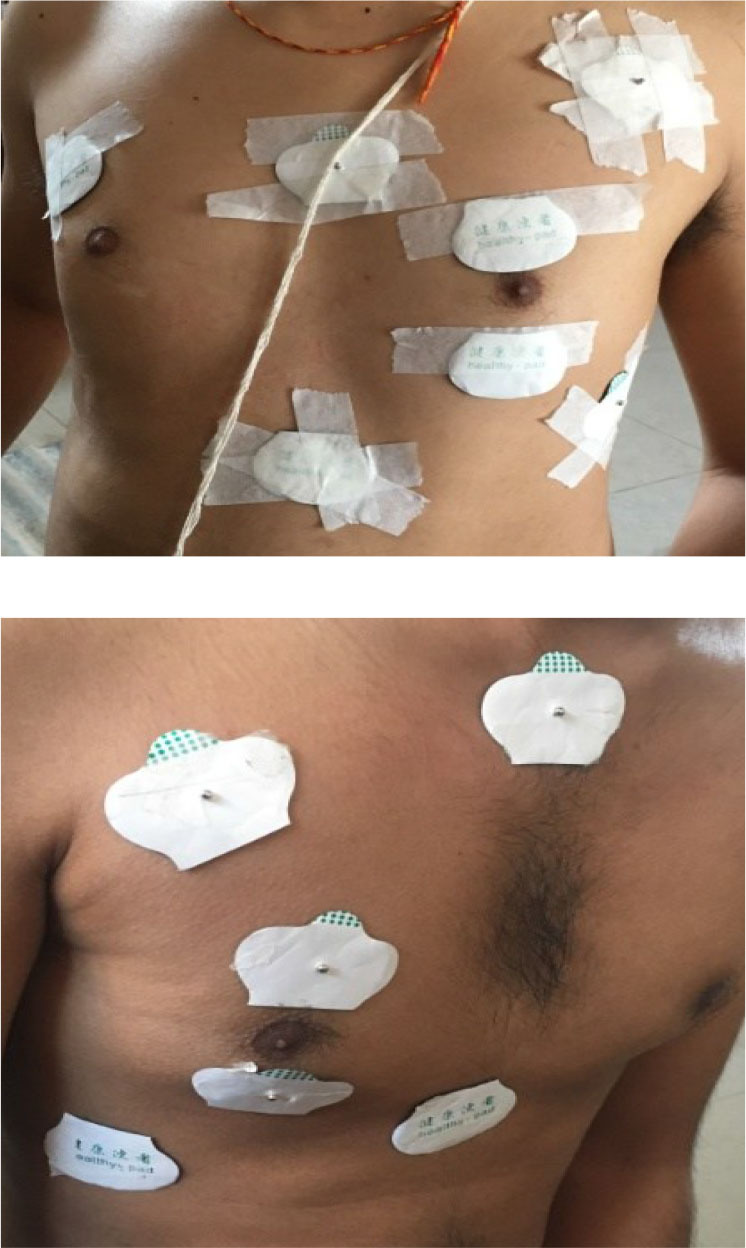
Electrode placement for measurement at the lower left lung (top) and the lower right lung (bottom).

**Fig. 9:**
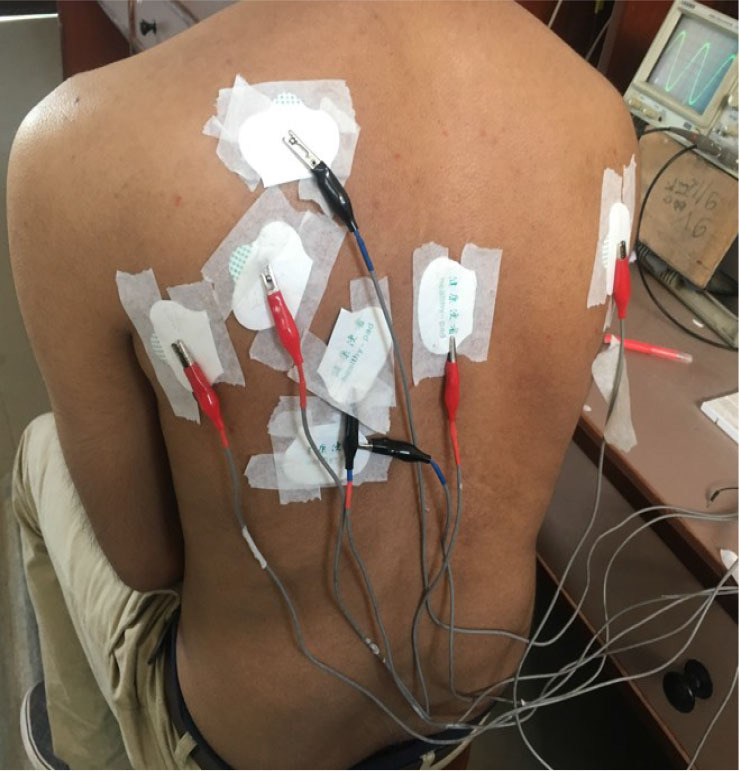
Electrode placement for measurement at the lower left lung from the back.

Measurements were also performed for left and right lungs, from front as well as from the back. Figures 8, 9 and 10 show some of these configurations. Measurements were taken with the subjects taking a deep breath (maximum inspiration) and then with the subjects breathing out as much as possible (maximum expiration). The sinusoidal voltage output obtained from the middle pair of potential electrodes, which essentially gives the focused transfer impedance was measured on the oscilloscope. Appreciable variation could be seen between breathe in and breathe out conditions.

For each electrode configuration, the difference was then expressed as a percentage change with respect to the value at maximum expiration. This is referred to as the percentage change of the focused impedance. Each measurement was repeated three times and averaged to minimize the effect of changing volume of blood in the heart.

The above measurements for lungs ventilation using the designed FIM-6 system were performed on three healthy male subjects, all non-smokers. Emphasis was given to the right lower lung since this region of the lung could give us the focused transfer impedance while eliminating the effects of heart movement. The right and left lungs were separately studied using the FIM-6 configuration.

FIM-6 studies on the front of the thorax were made on two of the three healthy male subjects. Instructions were given for inspiration and expiration. The electrodes for FIM-6 configuration on the body were placed as closely as possible to match the requirements as shown in Figures 7 and 8 where the nipple positions were chosen as reference points for the placement of the electrodes. The initial separation between the potential electrodes was fixed at 9.0 cm with the nipple position in the middle as in Figure (8).

Measurements were also taken by shifting the electrodes' positions with the arrangement as in Figure 8 (bottom).

The current electrodes were raised and lowered by 2 cm, with the position of potential electrodes unchanged at first and then each potential electrode was also raised and lowered by 2 cm to perform the experiment.

The third subject underwent FIM-6 studies on the back of the thorax. Electrodes were placed to study the left lower lung, as shown in Figure 9. This was done to minimize the effects of heart movement. The process for inspiration and expiration was repeated five times to minimize the errors.

### Ethical approval

The research related to human use has been complied with all relevant national regulations, institutional policies and in accordance with the tenets of the Helsinki Declaration and has been approved by the authors’ institutional review board or equivalent committee.

## Results

### Linear TPIM arrangement

The gain of the designed non-inverting amplifier as shown schematically in Figure 3, was measured at a frequency of 10 kHz and was found to be 95.49 with the use of transformer 1 (TR1) across the current drive output.

As mentioned before, the current electrode positions were kept fixed with separations of 30.0 cm between electrodes of each orthogonal pair. Measurements were carried out for two separations of the potential electrodes, 10.0 cm and 12.5 cm, respectively. For the 10-cm separation between potential electrodes, the voltage drops across the potential electrodes placed on the body were noted as 7.96 millivolts and 6.28 millivolts for the maneuver of breathe in and breathe out, respectively. The value of the true current was noted as 0.47 mA. Thus, the value of the transfer impedance was calculated as 16.93 ohms and 13.36 ohms for breathe in and breathe out, respectively, contributing to a change of 26.72% with respect to that at breathe out. Similarly, for the 15-cm separation between the potential electrodes, the voltage drops across the potential electrodes for this arrangement were noted as 10.7 millivolts and 8.7 millivolts for the maneuver of breathe in and breathe out, respectively, corresponding to transfer impedance values of 22.7 ohms and 18.51 ohms, respectively. The change was 19.23%.

### FIM-6 arrangement for Lungs Ventilation Study

The results obtained during the measurement for the study of all three human subjects are shown below in comparative form. The outputs were measured from the sinusoidal waveforms on an oscilloscope.

### Subject 1

Placement of potential electrodes: Around the lung region (Figure 7). Comparison basis: Distance between potential electrodes in cm (Table 1). Measurement view: Front.

**Table 1:** Reading for the percentage change in impedance for different separation of potential electrodes for FIM-6 during lung ventilation. The first column shows the separation between the potential electrodes in cm. The second and third column show the FZ values for breathe in and breathe out, respectively, in ohms. The last column shows the percentage change in impedance with the value on breathe out as the reference.

**Separation between potential electrodes (cm)**	**Breathe in impedance (ohms)**	**Breathe-out impedance (ohms)**	**Percentage change (Ref: breathe out)**
10.0	21.30	18.89	12.76
12.5	28.98	24.63	17.66

### Subject 2

Placement of potential electrodes: Lower region of the lungs as shown in Figure 8 (top) and (bottom). Comparison basis: right and left lung placement (Table 2). Measurement view: Front.

**Table 2:** Reading for the percentage change in impedance for arrangement of FIM-6 electrodes on the right lung and left lung during lung ventilation. The first column shows the arrangement scheme for the electrodes in the lung region. The second and third column shows the FTZ values for breathe in and breathe out in ohms. The last column shows the percentage change in impedance.

**Arrangement**	**Breathe in impedance (ohms)**	**Breathe out impedance (ohms)**	**Percentage change (Ref: breathe out)**
Lower right lung	29.52	25.32	16.58
Lower left lung	19.13	15.19	25.90

### Subject 3

Placement of potential electrodes: Lower region of the lungs. Comparison basis: Right and left lung placement, shifting of current and potential electrodes by 2 cm each (Table 3). Measurement view: Front for right lung (Figure 8 bottom) and back for left lung (Figure 9).

**Table 3:** Reading for the percentage change in impedance for arrangement of FIM-6 electrodes on right and left lung during lung ventilation. The first column shows the study region. The second column shows the arrangement scheme of electrodes for FIM-6 configuration. The third and fourth column shows the FZ values for breathe in and breathe out in ohms. The last column shows the percentage change in impedance.

	**Arrangement**	**Breathe in impedance (ohms)**	**Breathe out impedance (ohms)**	**Percentage change (Ref: breathe-out)**
Lower right lung	Initial	15.35	12.87	19.27
	Shifting of current electrodes only	12.38	9.90	25.05
	Shifting of both current electrodes and potential electrodes	27.23	21.29	27.90
Left lung	Back part of the left lung	18.56	16.09	15.35

## Discussion

The present version of FIM-6 was also employed for a lung ventilation study in a simultaneous manner with the linear TPIM arrangement of carbon coated electrodes. In case of linear TPIM, on increasing the separation between the potential electrodes, the focused transfer impedance values for breathe in and breathe out increased. With the increase in the separation between potential electrodes, a larger volume within the lung region contributed to the transfer impedance, hence leading to the increment. However, the reduction in percentage change for the 15-cm separation is probably due to the influence of negative sensitivity contributing to the uncertainty. The study using the FIM-6 version shows that on increasing the separation between the potential electrodes the focused impedance values for breathe in and breathe out increased and the percentage change in impedance values also increased. Impedance increases for greater separation between the potential electrodes as the effective length of the measured region increases, i.e. R ∝ L/A.

The increase in percentage change can be attributed to the following points:

a) Within the total sensitive volume, more lung regions contributed to total FZ (FZ constitutes a fixed value from non-lung regions + variable value from lung regions).
b) The heart position changes between breathe in and breathe out, which may also contribute to this increase.
c) Negative sensitive zone is greater for the 10-cm separation of potential electrodes compared to that for the 12.5-cm separation (since current electrode separation is constant), this effectively reduces the percentage change in the 10-cm case.

For subject 2, the focused transfer impedance on the left is less than that on the right. This is possibly due to the presence of the heart with the large amount of blood in it. The percentage change in impedance is high for the left lung in comparison to that of the right lung, which is not expected but it is possibly due to geometrical positioning of the heart.

For subject 3, the results are interpreted in the following points:

a) In arrangement 2, current density under the potential electrodes decreases, (current spreads out over a greater volume) so the measured potential (V = I × Z) decreases. On the other hand, in calculating FZ, we use the same constant current driven into the body, so FZ (=V/I) decreases.
b) In arrangement 3, the potential electrode separation increased, so measured potential (V = I × Z) is increased.
c) In arrangement 2, current goes into a deeper region, hence including more of the lungs in the sensitive volume, and the percentage change is increased.
d) In arrangement 3, there will be a smaller zone with negative sensitivity, compared to that in 2. Therefore, the percentage change is higher (although 25% and 27% may not be that significant)
e) The back side has a thick muscle between the skin and lungs. Hence, there is less contribution from the lung, so the percentage change is less compared to the frontal measurement on the right lung.
f) All the organs in the thorax, including the lungs and heart, move vertically during breathing. Hence, in the Impedance measurements using electrodes with large separation, there would be a contribution to the changes due to movement of the organs. So not all the changes are due to lung ventilation alone.

When we describe “True Load Current” as twice the current through each transformer, this nomenclature also needs to be discussed. The two currents are driven from two orthogonal directions, so they do not add up everywhere. Possibly we should use the phrase “Sum of the two currents”.

The FTI on the left is less than that on the right. Possibly this is due to the presence of the heart with the large amount of blood in it. Since blood volume changes over a heart cycle (about 1 sec), several readings were taken.

We cannot still rule out the effect of the change in the internal organs in between the two measurements. In fact, this is one of the greatest challenges of impedance measurements. People have tried to measure Cardiac Output using electrical impedance but could not isolate that due to changes in heart position within the heart cycle. The sensitivities of the outer electrodes diminish very rapidly with depth. We have tried to use this concept in the present studies.

The six-electrode FIM system does not require a computer or microcontroller, which makes it useful in real life for physiological studies with little sophistication. In the present work, the output was measured using a sinusoidal signal from an oscilloscope. In practical systems, a voltmeter could be used for numerical outputs with the necessary modifications in the instrumentation.
